# On the Mechanical Properties of Hybrid Dental Materials for CAD/CAM Restorations

**DOI:** 10.3390/polym14163252

**Published:** 2022-08-10

**Authors:** Teresa Palacios, Sandra Tarancón, José Ygnacio Pastor

**Affiliations:** 1 Department of Geological and Mining Engineering-CIME, Universidad Politécnica de Madrid, 28003 Madrid, Spain; 2 Department of Materials Science-CIME, Universidad Politécnica de Madrid, 28040 Madrid, Spain

**Keywords:** fracture analysis, micro-structure, electron microscopy, CAD/CAM, hybrid materials

## Abstract

Two hybrid dental materials available for computer-aided design and manufacturing (CAD/CAM) dental restorations have been selected to explore their potential. On the one hand, the scarcely investigated polymer-based material Vita Enamic^®^ (VE) and, on the other hand, the leucite-based material IPS Empress^®^ CAD (EC). Their micro-structure and mechanical performance were analyzed in two environments: directly as received by the manufacturer (AR), and after immersion and storage in artificial saliva (AS) for 30 days to determine the influence of the saliva effect. To avoid an inappropriate selection of materials for clinical use, a full understanding of their mechanical behavior is essential. Therefore, this investigation aims to determine the micro-structural and chemical composition by field emission scanning electron microscopy (FE-SEM) and X-ray fluorescence analysis, establishing the density, micro- and nano-hardness, the nano-elastic modulus, and the flexural strength and fracture toughness (by introducing a femto-laser notch to replicate a real crack). In addition, fracture surfaces of the broken samples were analyzed to correlate the failure micro-mechanisms with their mechanical properties. Results indicate that while the crystalline phase of the materials is very similar (composed of SiO_2_ and Al_2_O_3_), the micro-structure and mechanical behavior is not. The material EC, with finer micro-structure, exhibits a higher mechanical performance but with greater variability of results. Furthermore, the material VE, with a 25 vol.% polymer phase, shows a mechanical performance similar to enamel and dentin and therefore more similar to human behavior.

## 1. Introduction

Dental materials for CAD/CAM systems have traditionally been divided into ceramic-based materials and composite-based materials [1]. The first category covers most clinical indications due to their biocompatibility, wear resistance or low thermal conductivity; however, they exhibit low tensile strength and high brittleness, which are fundamental for their predominant structural applications [2,3]. Remarkably, within the broad selection of ceramic-based materials, the feldspathic ceramics evolve to become more resistant materials by the addition of leucite crystals (35–50 wt.%) in the vitreous matrix, which improve their mechanical properties without compromising the optical characteristics [4]. Conversely, composite-based materials are becoming good alternatives due to their excellent mechanical and physical behavior and potential to generate additional functionality, due to factors such as their antimicrobial and good aesthetic properties [5,6,7,8]. In addition, the processing characteristics of polymers, which can significantly simplify clinical procedures, allow them to be used in lower thickness than ceramic materials [9,10]. However, there is little independent evidence of their mechanical performance, unlike the already well-known and scientifically proven dental ceramics which define the accepted standards. Therefore, they are usually compared with them. In addition, the classification of the polymer materials in groups is not easy since materials can vary due to monomer composition, chemical composition, filler size or arrangement [11].

Another vital parameter to consider in developing new materials is their similarity to natural teeth. The restoration must not only fulfill the mechanical requirements, but also the optical ones, and be imperceptible. They should be manufactured in an adequate range of dentin and enamel, the main components of natural teeth structure [12]. The outer layer consists of enamel, the most complex and challenging component of the tooth composed of 96% hydroxyapatite and 4% organic substances and water [13]. The bulk of the tooth consists of dentin, which is softer and is composed of 45 vol.% minerals mainly hydroxyapatite, up to 30 vol.% organic material collagen and 25 vol.% water [14]. Furthermore, the center of the tooth is occupied by living tissue called the pulp.

Newly developed hybrid materials, therefore, are expected to combine the proven mechanical performance of ceramics with those of the composite materials for CAD/CAM technology which will provide considerable benefits for the user [15]. This study evaluates the physical, micro-structural, and mechanical properties of two hybrid materials: a polymer-based dental material (Vita Enamic^®^, VE) and a leucite-based material (IPS Empress^®^ CAD, EC). The findings were compared with results from other authors and the previously studied and widely extended commercial resin-based materials such as Lava^TM^ Ultimate (LU) or Cerasmart^TM^ (CS). With all this data, it will be possible to determine the behavior most suited to the actual properties of the available materials for dental restorations, since the information provided by manufacturers is in many cases poorly referenced or simply unavailable. The final aim of this work is to obtain an accurate, complete, and objective technical knowledge about the materials to enable a proper selection by dental personnel.

## 2. Materials and Methods

### 2.1. Materials and Specimen Preparation

Among the broad number of restorative CAD/CAM commercial materials currently available for indirect dental restorations, two hybrid materials were selected: Vita Enamic© (VE) and IPS Empress^®^ CAD (EC). Their technical information as exposed by manufacturers is shown in Table 1 [15,16].

**Figure 1:**
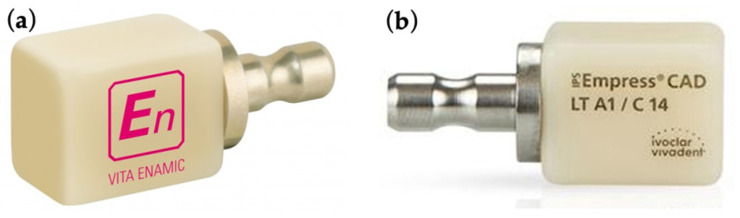
Chairside CAD/CAM C14 blocks: (**a**) VE and (**b**) EC.

The starting materials were CAD/CAM C14 blocks with approximate dimensions of 18 × 16 × 18 mm^3^ (Figure 1). These materials are brittle so, to obtain the test specimens, blocks were embedded in epoxy resin and cut-off in a two-step process with an Accutom 50 (Struers, Ballerup, Denmark). The process was performed under standard laboratory conditions at room temperature (22 ± 2 °C) and 50 ± 10% relative humidity. The cutting was performed by using a diamond disk under high-flow-water refrigeration; first into slices of 1.5 mm thickness and subsequently embedded against and cut into their nominal miniaturized beam dimensions of 1.5 × 1.5 × 17 mm^3^. Before being used, the beam specimens were cleaned in distilled water for 10 min by ultrasounds.

The miniaturized beam specimens were used to perform all tests except for fracture toughness. In this case, a notch was introduced using ultra-short laser ablation, so-called single edge laser-notch beam (SELNB) method [17]. This technique produces very sharp notches in brittle materials which are comparable to a crack introduced by fatigue with accuracy and good reproducibility. As demonstrated by previous studies for brittle metals [18,19] and dental materials [20], it provides a real measure of the fracture toughness in brittle materials with reliable results.

### 2.2. Ageing Procedure

Half of the analyzed specimens were immersed and storage in artificial saliva (BZ109 Biochemazone, Alberta, Canada) to analyze its influence on the mechanical properties. The AS was used at room temperature for 30 days, which was demonstrated to be enough time to stabilize the degradation of the properties [21]. Aged samples and as received by the manufacturer (AR) were tested under the same conditions and compared with the technical data from manufacturer (MN) and results from other authors.

### 2.3. Micro-Struture and Fracture Surface Characterisation

After the failure of random specimens of each material, the micro-structure and the fracture surfaces were examined with an Auriga column field emission scanning electron microscope (FESEM) from Zeiss (Oberkochen, Germany). To analyze the micro-structure, the samples were embedded in epoxy resin for grinding and polishing. After that, they were etched with 2% HF during 5 s to reveal the micro-structure and coated with Cr. Then, they were cleaned with ethanol immersion to analyze the morphology and grain topography of the post-mortem fracture surfaces.

Furthermore, the micro-structural surfaces of VE obtained with the FESEM were analyzed using the IMAGE-J software. The images were initially preprocessed to improve the signal-to-noise ratio, increasing their contrast, and then converted into binary. Subsequently, with segmentation, the different regions of interest (ROI) were delimited, and the quantification was accomplished using these zones to evaluate the percentage of each of the two existing phases in the material: the polymeric and the ceramic. To obtain valid results, 15 images were taken at different magnification and from various areas, thus ensuring that a representative sample of the material was observed. The data obtained from quantifying each phase, and disregarding the possible residual porosity, were indicated.

### 2.4. X-ray Fluorescence Analysis

The elemental composition was obtained with X-ray fluorescence (XRF) spectrometry. In this non-destructive technique, tiny samples of each material, previously cleaned with ethanol are introduced in a Zetium XRF spectrometer from Malvern Panalytical (Egham, Surrey, England) to identify the crystalline phases and the chemical elements through semi-quantitative analysis.

### 2.5. Density Measurement

The Archimedes method was used to measure the experimental density of the materials in the AR-state. The measurements were performed with a Mettler Toledo AG245 (Columbus, OH, USA) by immersion in high purity ethanol at 22 °C and a mass with a resolution of 10^−4^ g. At least six samples of each material were measured.

### 2.6. Vickers Tests

Micro-indentation tests with a Vickers indenter were one method used to calculate hardness. AR and AS samples were tested at room temperature with a durometer AKASHI MVK-EIII (Osaka, Japan) and an applied load of 9.8 N for 12 s by following ASTM E92–27 [22].

### 2.7. Nano-Indentation Tests

Instrumented nano-indentation tests were the second method used to measure hardness. Tests on AR and AS samples, were performed with a NanoIndenter XP from former MTS Systems Corporation (Minnesota, United States) and a standard Berkovich tip calibrated with fused silica at room temperature and under load control with an applied load of 0.25 N and measuring the displacement. Based on the load-displacement data obtained, the average values (with their corresponding standard error) of nano-hardness were calculated according to the Oliver and Pharr method [23,24]. From these tests, the nano-elastic modulus was also calculated. This data, along with the fracture toughness, is a fundamental property of the material, representing the real bond between atoms.

### 2.8. Three-Point Bending Tests

Three-point bending (TPB) tests were performed in the miniaturized samples to measure the ultimate strength in bending, i.e., flexural strength, which is the most common method to determine strength in dental materials [25] and fracture toughness, which is a relevant property for dental applications that reveals the resistance of materials against crack propagation. This resistance to fracture in the presence of a defect along with the elastic modulus is a real indicator of the material´s resistance.

TPB tests were performed until failure in an Instron 3369 (Norwood, MA, United States) using displacement control at a constant crosshead speed of 100 µm/min and a load span of 12 mm following dental standards ISO 4049 [26] and ISO 6872:2015 [27], and a reproducible setup as described in [20] for specimens with dimensions 1.5 × 1.5 × 17 mm^3^. TPB tests were performed in at least six miniaturized samples without notch (smooth specimens) to obtain flexural strength. Based on the force-displacement linear curves obtained, the maximum bending strength was calculated at the fracture point by using the standard material strength formula [28]. In addition, these curves were used to calculate the elastic modulus was calculated under two conditions: AR and AS. The miniaturized SELNB specimens use the same setup to obtain fracture toughness. For each specimen, the initial notch length “a” was measured using a FESEM, and the maximum applied load was recorded during the test to calculate the fracture toughness using the appropriate formula [29].

## 3. Results

### 3.1. Composition and Micro-Structure

**Figure 2:**
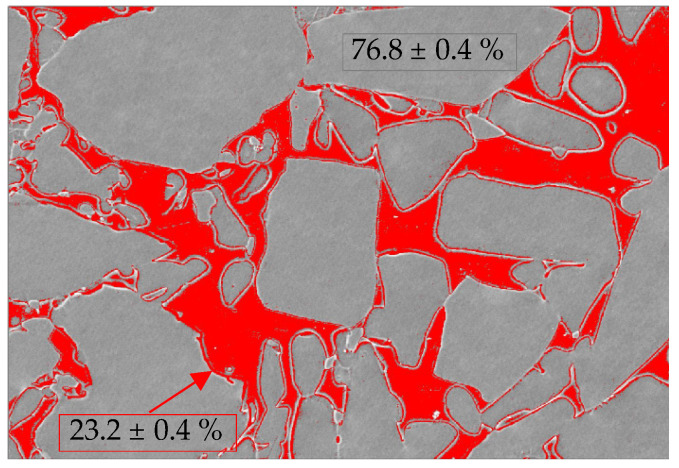
Image processing analysis obtained from IMAGE-J to quantify the area of each phase (in red the polymer area and in grey the ceramic area) for VE.

**Figure 3:**
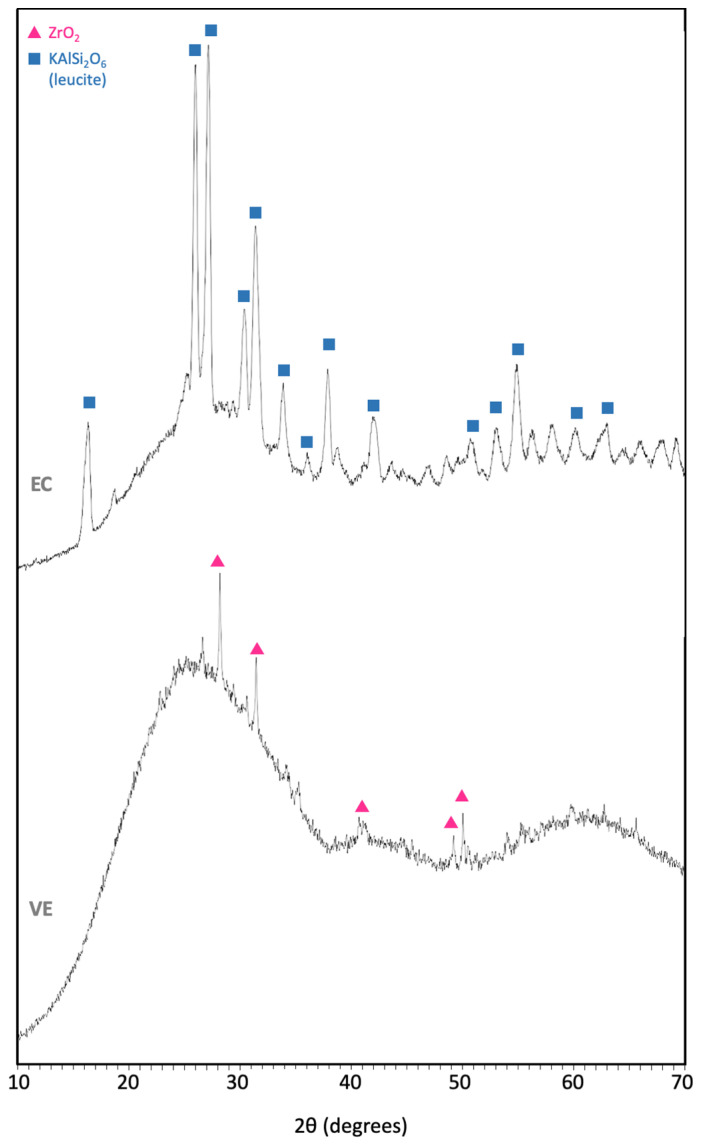
X-ray fluorescence (XRF) patterns of the crystalline phases of EC and VE.

The hybrid material VE is composed of a sintered ceramic structure matrix with a reinforced polymer network, which are fully integrated with one another [15]. The organic polymer content is 14 wt.%/25 vol.%, while the inorganic ceramic content is 86 wt.%/75 vol.% (Table 1), in accordance with the image processing from the analysis of several polished surfaces (Figure 2). The element concentration of the ceramic crystalline phase exhibited from the XRF analysis is between the range exposed by MN; mainly composed of SiO_2_ enriched with Al_2_O_3_ (Table 2). Crystalline ZrO_2_ (Figure 3) is also noticeable from the XRF patterns, which is the only crystal phase that was possible to detect.

Secondly, the EC is composed of leucite (35–45 vol.%) reinforced with glass ceramic displaying amorphous and crystalline phases. The main constituents of this latter phase are also SiO_2_ and Al_2_O_3_, and some other oxides revealed by the XRF analysis which are in a range in accordance with MN data (Table 2). From de XRF patterns for the crystallized phases (Figure 3), only leucite crystals (KAlSi_2_O_6_) were revealed for EC.

**Figure 4:**
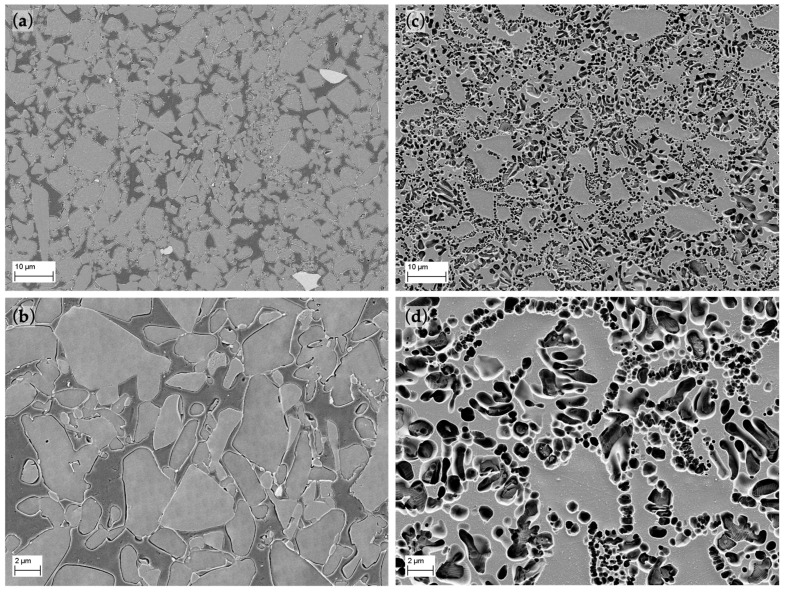
FESEM micrographs of polished and etched surfaces of (**a**,**b**) VE and (**c**,**d**) EC materials.

**Figure 5:**
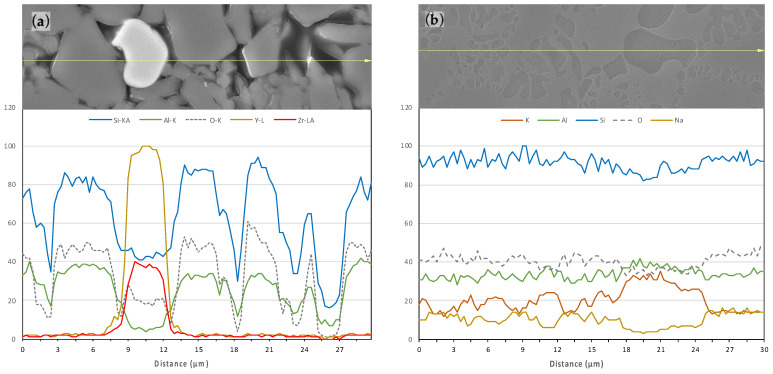
FESEM micrograph of a polished surface with its EDX line-scan analysis of (**a**) VE, and (**b**) EC.

After the first etching of EC the glassy phase was partially dissolved, but after several attempts it was possible to reveal the micrometer-sized ceramic grains embedded in the polymer matrix. The size distribution of the grains ranges roughly from 1 to 20 µm (Figure 4a,b). The micro-structure consists of one continuous ceramic network (grey areas) and a polymer network (dark areas). Some marginal light grey particles can be distinguished and distributed along the surface. An EDX line scan analysis was performed (Figure 5) to determine the precise composition of those areas, defining them as Y- and Zr-rich particles, whereas Si and Al particles (SIO_2_ and Al_2_O_3_) are distributed all over the surface (grey polyhedral grains).

Although the chemical composition of the crystalline phase is very similar for both materials, the VE micro-structure is somewhat different (Figure 4c,d). It is composed of a light grey network that surrounds darker areas with rounded and elongated shapes with sizes up to 5 µm. These empty darker areas were the space of the leucite crystals dissolved by the etchant as revealed by the EDX line scan analysis (Figure 5b).

### 3.2. Density

For both materials, the measured density (mean ± standard error) in the AR-state is 2.08 ± 0.01 g/cm^3^ for EC and 2.435 ± 0.001 g/cm^3^ for EC, which is in the range of the one provided by the manufacturer 2.1 ± 0.1 g/cm^3^ for VE [15] and 2.5 ± 0.1 g/cm^3^ for EC [30]. From FESEM images (Figure 4), materials reach a high densification rate since no significant pore areas are observed.

### 3.3. Micro- and Nano-Hardness

**Figure 6:**
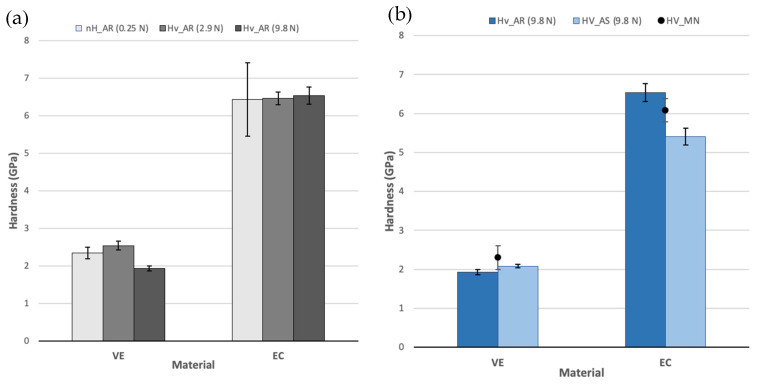
Micro- and nano-hardness (mean and standard error) for VE and EC (**a**) Influence of the applied load and the test method on AR specimens; (**b**) influence of the saliva immersion (AS) and the comparison with the manufacturer (MN) data.

In general, VE exhibits a considerably lower hardness than EC, which is in the range of 2 GPa, meanwhile for EC it is in the range of 6 GPa, almost three times harder. Materials tested in air under various loads (Figure 6a) exhibit a marginal size effect on the results; only in the case of VE a slight decrease of 20% is observed for tests performed at the highest load. These results in the AR-state are comparable with values reported by MN (Figure 6b) and other authors [30,31].

The influence of the AS (Figure 6b) is negligible for VE exhibiting values in accordance with MN performed with an applied load of 30 N for 20 s [15]. However, this effect is not insignificant for EC where the material experiences a significant detriment of its hardness of around 20%.

### 3.4. Elastic Modulus

**Figure 7:**
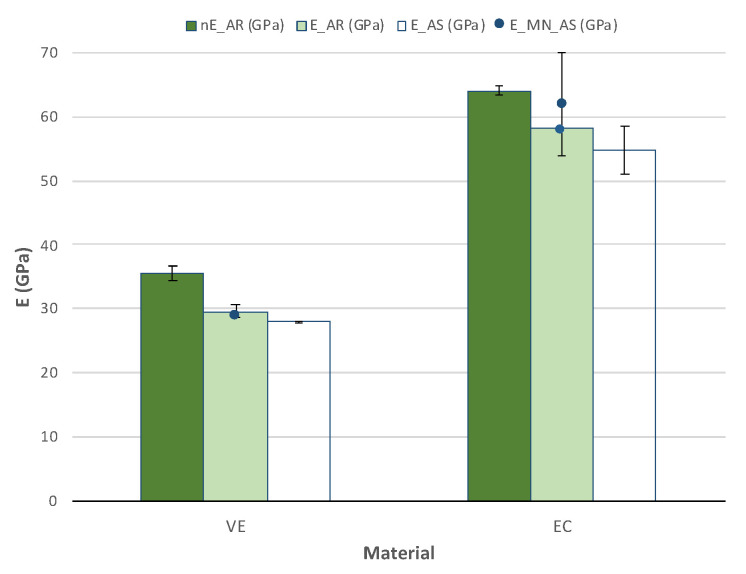
Elastic modulus (mean and standard error) was obtained from nano-indentation tests (nE), TPB tests (E) in the AR and AS states and data from the manufacturer (MN).

Various methods calculated the elastic modulus (Figure 7). The first was from the impulse excitation technique (IET), the second from the nano-indentation tests and the third from the load-displacement curves obtained in the TPB tests. The other two methods show values around 30 GPa for VE and double that, around 60 GPa, for EC. Unfortunately, results from the first method must be discarded since they were inconsistent due to the miniaturized specimen’s size as demonstrated in previous studies [20].

Nano-indentation hardness exhibits slightly higher values for both materials. Some authors, however, were able to determine elastic modulus with the IET technique with reported values around 37 GPa for VE [32,33] and 65 GPa for EC [32,34], in the range of the nano-indentation results.

Values obtained from force-displacement curves are in the range of those exhibited by the MN, although the force applied was 30 N instead of 9.8 N, especially for VE. Therefore, a slight influence of the applied load should be considered since nano-indentation values are overestimated. No effect of the AS immersion was detected in any of the materials.

### 3.5. Flexural Strength

**Figure 8:**
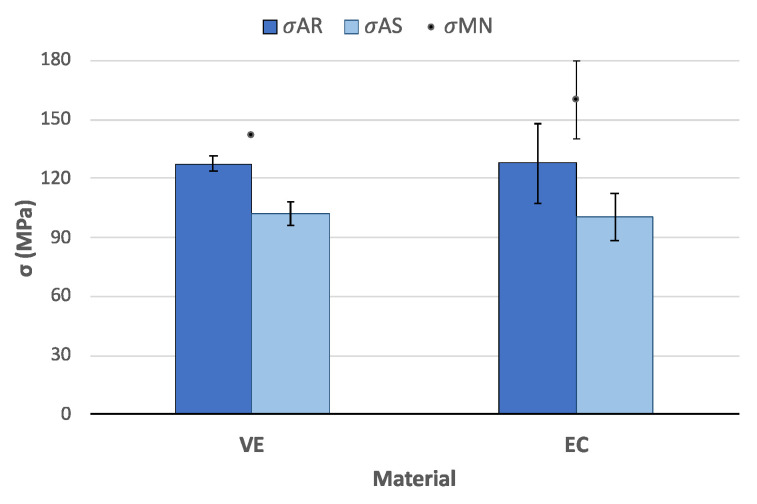
Flexural strength (mean values and their corresponding root mean square errors) after the TPB tests on unnotched samples of the materials AR and after immersion in AS and results from the MN.

Results from TPB tests on the smooth specimens (Figure 8) exhibit very similar results of flexural strength for both materials. They present values of around 125 MPa in the AR-state and influence of the saliva with a decrease of about 20%. Values reported by MN are very variable, particularly in the case of VE with a flexural strength of 142 MPa [35], which is an overestimation of around 10% in comparison to the AR-state. The influence of the saliva immersion is similar for both materials as well, with a loss of flexural strength around 20%, although in the case of VE, which exhibits much higher dispersion of results, the values are overlapped.

### 3.6. Fracture Toughness

**Figure 9:**
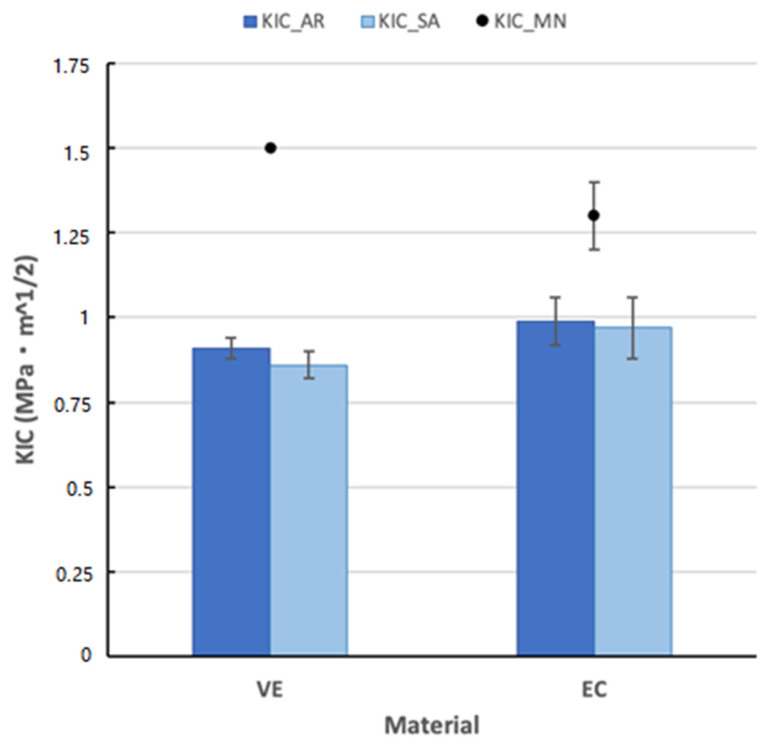
Fracture toughness (mean values and their corresponding root mean square errors) after the TPB tests on the SELNB samples of the materials AR and after immersion in AS and results from the MN.

Results obtained from TPB tests on the SELNB specimens (Figure 9) show low fracture toughness values, under 1 MPa⋅m^1/2^. About 60% higher fracture toughness values for VE [15] and 30% higher values for EC [30] were reported. In the AR-state, values for EC are about 9% higher than those for VE. Although, as in the case of flexural strength, they exhibit higher dispersion of results as well. Nevertheless, for this property, there is a large disagreement with results documented by MN in comparison to AR tests.

A slightly decrease in fracture toughness is observed with the AR immersion, however, there is also a high variation of results here.

### 3.7. Fracture Surfaces

**Figure 10:**
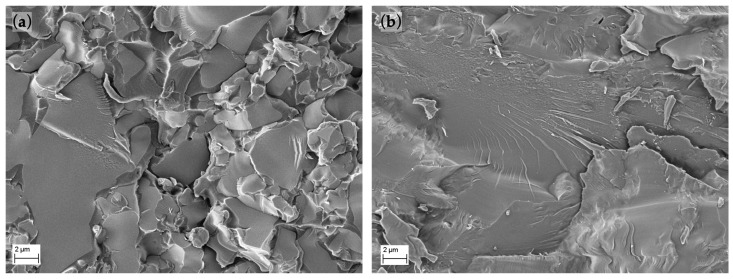
Representative fracture surfaces of: (**a**) VE; (**b**) EC after a TPB test of the materials AR.

In the macroscopic scale, both materials exhibit flat breakage on the plane perpendicular to the applied load without any sign of plastic deformation. In the microscopic level (Figure 10) the brittle fracture can also be noted with the grain’s flat breakage. No significant differences were found between fracture surfaces in samples AR and after immersion in AS.

## 4. Discussion

This study investigates the micro-structure, composition, and mechanical properties of two hybrid materials for CAD/CAM dental restorations: VE and EC. Tests were performed under two environments: directly AR by the manufacturer and after 30 days storage and immersion in AS. The fracture surfaces after breakage were also analyzed to correlate the mechanisms of failure involved and the micro-structure and composition with their mechanical behavior.

Both materials contain a ceramic phase, but the hybrid VE also contains a 25 vol.% polymer phase with a composition consisting of UDMA and TEGDMA (Table 1). This is a simpler mixture as opposed to other dental polymers such as previously studied Lava^TM^ Ultimate (LU) or Cerasmart^TM^ (CS) [20]. The EC material is only composed of a ceramic matrix reinforced with 35–45 vol.% leucite. Although the crystalline phase of both materials determined by XRF analysis (Table 2) has an analogous composition, mainly composed SiO_2_ and Al_2_O_3_, their micro-structure (Figure 4) is diverse. The analysis of the polished surfaces reveals a more refined particle size distribution with rounded leucite grains up to 5 µm embedded in the ceramic matrix for EC. In contrast, VE shows a coarser size distribution with polyhedral grains between 1 to 20 µm and some Y- and Zr-rich grains up to 10 µm, all surrounded by a polymer matrix. This is a coarser micro-structure than compared to other polymeric materials [20], although with similar morphology and insignificant porosity.

Measured density values in the AR-state shows a 15% lower density for VE, with values of around 2.1 g/cm^3^ for VE and 2.5 g/cm^3^ for EC. Both results are following MN reported values [15,30] and other authors [32,33]. The density of VE is similar to other dental polymeric materials, although its composition differs [20]. The densification rate of both materials is suitable since there is no evidence of significative porosity in the analyzed micro-structure (Figure 4).

The micro-structure is well defined into leucite crystals and ceramic matrix, and both phases have heterogenous properties. Although VE exhibits lower hardness values than EC, in the range of 2 GPa and maybe related to its polymer content, they are between the values of dentin (0.6–0.92 GPa [36,37]) and enamel (3–5.3 GPa [38,39]). This fact favors the proper behavior of the alloy, since its performance will be more similar to the original human materials. In addition, this hybrid material, VE, exhibits higher hardness than other polymerics such as LU (1 GPa) or CS (0.8 GPa). In addition, it shows no significant influence on the AS immersion, meaning that properties will not be reduced when the material is in mouth. EC, meanwhile, displays a hardness around 6 GPa with a higher dispersion of results that may be due to differences in the indentation point. This material also exhibits an important detriment of its hardness values when it is in continuous contact with the AS.

Several methods calculated the elastic modulus, and the most accurate values were obtained from the analysis of the force-displacement curves from TPB strength tests since the whole specimen is considered and considered all possible defects present in the material [20]. A slight size effect on the indentation load was detected, but results with the 9.8 N load exhibit the most accurate elastic modulus. The obtained values are higher (around 60 GPa) for EC, double those of the VE material (around 30 GPa). In this case, although VE elastic modulus is within the range of human dentin, 26.97 ± 1.07 GPa [15,40], EC is found between dentin and enamel, with values reported as roughly 70–100 GPa [39]. The immersion in AS does not affect the elastic modulus.

Despite the grain size distribution of VE being much coarser than that of EC, there is no significant difference in the flexural strength resistance of the materials in the AR-state. Both exhibit values of about 125 MPa and a similar influence of the AS immersion with a detriment of 20%. However, although the obtained values are slightly lower than for other polymeric materials referred to above, such as LU with 20% higher flexural strength [20], they are within the range of dentin flexural strength. The resistance of the human dentin strongly depends on the specific piece and is significantly lower with the patient´s age [41]. Reported values range from 114 ± 36 MPa to 274 ± 97 MPa, although the most commonly reported are around 200 MPa [42]. There is also a disagreement between values reported by MN and other authors with an increase of 10% for VE [35] or even higher for EC with an overestimation of 25% and variable values of even 160 MPa [16,30]. This dispersion of results may be due to the variety of setups applied to perform tests that sometime are not well defined.

Fracture toughness values are in the range of 1 MPa⋅m^1/2^, although results for EC are 9% higher, both are in the range of other polymeric dental materials [20], which exhibit the same brittle behavior. Obtained values are between human enamel which ranges from 0.6–1.5 MPa⋅m^1/2^ [36,39], and human dentin with values from 1.8 to 3.1 MPa⋅m^1/2^ [36,43,44]. As shown in Figure 9, a huge overestimation of results reported by MN (30% for EC and 60% for VE) may come from differences in the notch root radios of the notches since the performing conditions of such tests are not specified. Therefore, they may use coarser notch root radius such as single edge V-notch beam specimens (SEVNB) which can lead to an overestimation of values as demonstrated in previous works for brittle materials [17,18,19] instead of SELNB (as explained in Section 2.8) which introduce notches similar to a real crack, so the real fracture toughness is measured. The AS immersion does not influence fracture toughness behavior.

Fracture surfaces exhibit brittle characteristics on the macro- and microscopic scale with a morphology. Values which are also in accordance with the TPB test results where stress–strain curves were lineal until fracture. The fracture toughness results show very low values corresponding to brittle materials.

## 5. Conclusions

The following conclusions of this investigation can be drawn:

1. Although results for XRF analysis exhibit similar composition of the crystalline phase for both materials (mainly composed of SiO_2_ and Al_2_O_3_), the micro-structure is dissimilar. EC exhibits a smaller grain size distribution. There is no evidence of significative porosity on the materials.
2. The hybrid material VE presents a 25 vol.% polymer phase, and although it exhibits a coarser micro-structure, and generally a lower mechanical performance, its mechanical properties have a better approach to human dentin and enamel.
3. The mechanical properties of EC exhibit higher density, hardness (about 6 GPa) and elastic modulus (about 60 GPa), but very similar flexural strength (around 125 MPa) and fracture toughness (under 1 MPa⋅m^1/2^). However, it exhibits a higher dispersion of results too.
4. The most precise and representative values for the elastic modulus measurements were obtained from the force-displacement curves from TPB strength test. Size effect was observed in the indentation tests, but values with 9.8 N load are the most accurate ones.
5. Measured flexural strength and fracture toughness show significantly lower values than those advertised by manufacturers. However, the information they provide is normally incomplete and poor, so it should be related to the accuracy of the measuring devices.
6. Fracture surfaces show brittle macro- and micro-mechanisms of failure in accordance with the linear stress–strain flexural TPB curves and fracture toughness results, under 1 MPa⋅m^1/2^.
7. In general, both materials exhibit a good performance after the ageing process, which predicts maintenance of the materials’ mechanical properties inside the mouth. It was only observed a detrimental effect of 20% on the hardness of EC and the flexural strength of VE.
8. The technical data provided by manufacturers was found incomplete for some mechanical behavior properties. Specially results obtained for flexural strength and fracture toughness were far from the manufacturer’s report due to the improper test method.

## Figures and Tables

**Table 1:** Composition of the studied materials from their manufacturers’ technical documentation.

Material	Abbreviation	Manufacturer	Type
Vita Enamic^®^	VE	VITA Zahnfabrick, Germany	Polymer-infiltrated ceramic-network composed by 86 wt.%/75 vol.% porous ceramic network infiltrated by capillary action with 14 wt.%/25 vol.% polymer (UDMA, TEGDMA).
IPS Empress^®^ CAD	EC	Ivoclar Vivadent AG, Liechtenstein	Leucite (35–45 vol.%) reinforced glass ceramic

UDMA: urethane dimethacrylate; TEGDMA: trimethylene glycol dimethacrylate.

**Table 2:** Element concentration (%) of the crystalline phase determined with XRF analysis.

Material	SiO_2_	Al_2_O_3_	Na_2_O	K_2_O	BaO	ZrO_2_	CaO	Y_2_O_3_	Other
VE	61.47	21.47	7.97	7.54	-	0.62	0.35	0.25	0.53
EC	62.24	17.58	5.53	11.63	0.44	0.07	0.99	0.08	1.17

## Data Availability

Not applicable.
